# Optimizing nitrogen use efficiency in forest plantations: mechanistic insights from *Arabidopsis*, crops, and natural forestry ecosystems

**DOI:** 10.48130/forres-0025-0029

**Published:** 2025-11-26

**Authors:** Debin Qin, Ruqian Wu, Linlin Niu, Bo Jiang, Yiwei Li, Guohua Chai, Jie Luo, Xinmin An

**Affiliations:** 1 State Key Laboratory of Tree Genetics and Breeding, College of Biological Sciences and Technology, Beijing Forestry University, Beijing 100083, China; 2 National Engineering Research Center of Tree Breeding and Ecological Restoration, College of Biological Sciences and Technology, Beijing Forestry University, Beijing 100083, China; 3 Key Laboratory of Genetics and Breeding in Forest Trees and Ornamental Plants, MOE, College of Biological Sciences and Technology, Beijing Forestry University, Beijing 100083, China; 4 College of Biological Sciences and Technology, Beijing Forestry University, Beijing 100083, China; 5 College of Resources and Environment, Qingdao Agricultural University, Qingdao 266109, China; 6 National Key Laboratory for Germplasm Innovation & Utilization of Horticultural Crops, Hubei Hongshan Laboratory, Hubei Engineering Technology Research Centre for Forestry Information, College of Horticulture and Forestry, Huazhong Agricultural University, Wuhan 430070, China

**Keywords:** Nitrogen use efficiency, Forest plantations, Root morphology, Nitrogen absorption, Beneficial microorganisms, Nitrogen cycle

## Abstract

Forest plantations, such as poplar and eucalyptus, exhibit high nitrogen requirements that are vital for growth, biomass accumulation, and the production of high-quality timber. However, the investigation of nitrogen use efficiency (NUE) mechanisms in forest plantations lags far behind that in crops. In contrast, natural forest ecosystems, without chemical fertilizer inputs, demonstrate remarkable capacities for biological nitrogen fixation and internal nitrogen cycling. Drawing on nitrogen utilization strategies elucidated in *Arabidopsis*, crop species, and natural forest ecosystems, this review provides a comprehensive synthesis and proposes strategies to enhance NUE in forest plantations. Key approaches include optimizing root system architecture, increasing intrinsic nitrogen uptake capacity, and harnessing beneficial microorganisms to improve nitrogen utilization. Furthermore, the review highlights the promising opportunities for employing key regulatory genes and synthetic biology approaches to achieve targeted enhancement of NUE in forest plantations.

Forest ecosystems, covering approximately 30% of Earth's land surface, could capture 7.6 Gt of carbon dioxide (CO_2_) annually^[1,2]^. While the global forest area continues to decline, planted forests have expanded from 167.5 million hectares (ha) to 306 million ha from 1990 to 2022, now approaching 20% of the agricultural land area (1,573 million ha, FAOSTAT) (Fig. 1). Within planted forests, approximately 131 million ha are fast-growing forests that are intensively cultivated for productive purposes, such as *Populus*, *Paulownia*, *Pinus* and *Eucalyptus*^[3]^. These forests are also major sources of timber and non-timber products that are essential to human well-being^[4]^. Empirical evidence from poplar, eucalyptus, and loblolly pine demonstrated that moderate nitrogen fertilization markedly enhanced both photosynthetic rate and biomass production^[5−7]^. Consistently, low nitrogen could curtail stem biomass by 30%–60%, leaf biomass by 49%–68%, and CO_2_ assimilation rate by 34%–42% on six *Populus* genotypes in the greenhouse^[8]^. Given that forest plantations are typically established on nitrogen-deficient marginal lands, and successive rotations progressively deplete soil N, which requires 350–600 kg N ha^–1^ per cycle to avert land degradation, these plantations are confronting an intensifying nitrogen scarcity^[3,9]^. NUE, defined as the amount of biomass or product obtained per unit of nitrogen fertilizer supplied^[10,11]^ is therefore a critical trait for expanding plantation area and increasing timber yield.

**Figure 1:**
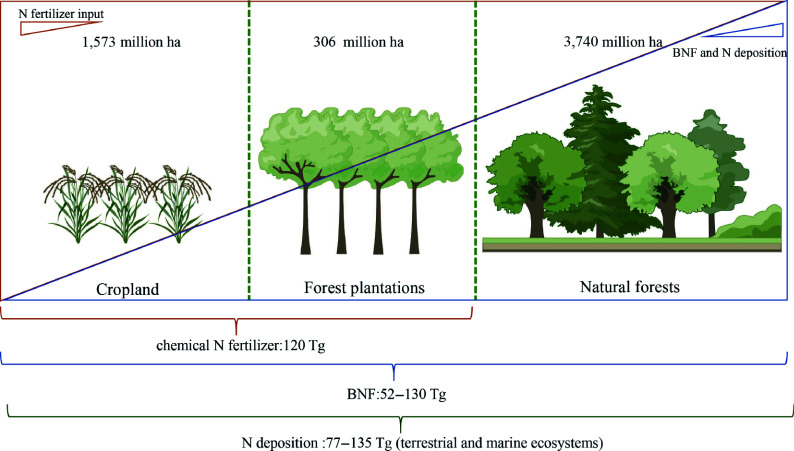
Nitrogen acquisition in agricultural and forestry ecosystems. The cropland area, planted forest area, and natural forest area can reach 1,573 million ha, 306 million ha, and 3,740 million ha, respectively. The primary modes of nitrogen acquisition in agricultural and forest ecosystems include chemical fertilizers, biological nitrogen fixation, and nitrogen deposition, which amount to approximately 120, 52–130, and 77–135 Tg (terrestrial and marine ecosystems) annually.

Terrestrial ecosystems acquire nitrogen sources via biological nitrogen fixation (BNF), chemical nitrogen fertilizer application, and nitrogen deposition. In agricultural system, up to 120 Tg (10^12^ g) chemical N fertilizer, comparable to the amount of nitrogen obtained through nitrogen deposition and BNF, was put into the cropland due to the continuous output of the biomass and low NUE^[12]^ (Fig. 1). Analogously, the productivity and profitability of short-rotation forest plantations depend on judicious N fertilisation^[13,14]^. Natural forests, in contrast, rarely require exogenous N fertilizer. Perenniality and long rotation lengths allow trees to develop deep root systems that exploit sub-soil N pools, while rhizosphere microbial activity mineralises organic N and mediates the uptake of deposited N, collectively satisfying up to 25% of tree N demand^[15−17]^. BNF further supplements the N budget, either through nodulation in leguminous taxa such as *Robinia pseudoacacia* or via associative diazotrophs in non-leguminous species such as *Populus*^[18,19]^.

Therefore, plant NUE is determined by a combination of genetic factors, such as root morphology, nitrogen uptake and assimilation capacity, and environmental factors, including soil nitrogen availability and the functional diversity of rhizosphere and phyllosphere microorganisms. These microbes influence NUE through BNF, participation in the nitrogen cycle, and plant growth promotion. Existing reviews have emphasised ecosystem-scale N fluxes and silvicultural management, but an integrated, breeding-oriented framework that couples host physiology, genomics, and microbiome function remains nascent. Translating mechanistic insights from *Arabidopsis*, major crops, and natural forests offers a roadmap for improving NUE in forest plantations. Here, three complementary strategies are synthesised: (1) regulating root system architecture (RSA) for enhanced tree NUE; (2) optimizing intrinsic plant pathways for inorganic nitrogen absorption and assimilation; and (3) leveraging microbial-mediated nitrogen cycling processes in the rhizosphere, with emphasis on BNF.

## Regulating root architecture for enhanced tree NUE

The root system constitutes the primary organ for nitrogen acquisition, and enlargement of the root-soil interface through increased length, reduced mean thickness, or lower tissue density constitutes a key determinant of elevated nitrogen-uptake efficiency^[20]^. Meta-analysis of 77 tree species shows that thinner roots or higher specific root length (SRL; root length per root dry mass) correlates with elevated nutrient-uptake capacity and enhanced substrate affinity. Under N limitation, proliferation of lateral roots (LR) and their elongation into nutrient-rich microsites dominate the foraging response^[21,22]^. RSA is thus a polygenic trait molded by intrinsic developmental programs and extrinsic cues (soil structure, N heterogeneity, and rhizosphere microbiota) that together determine the spatial deployment of absorptive surface area.

### Root system architecture in perennial trees and annual crops

**Figure 2:**
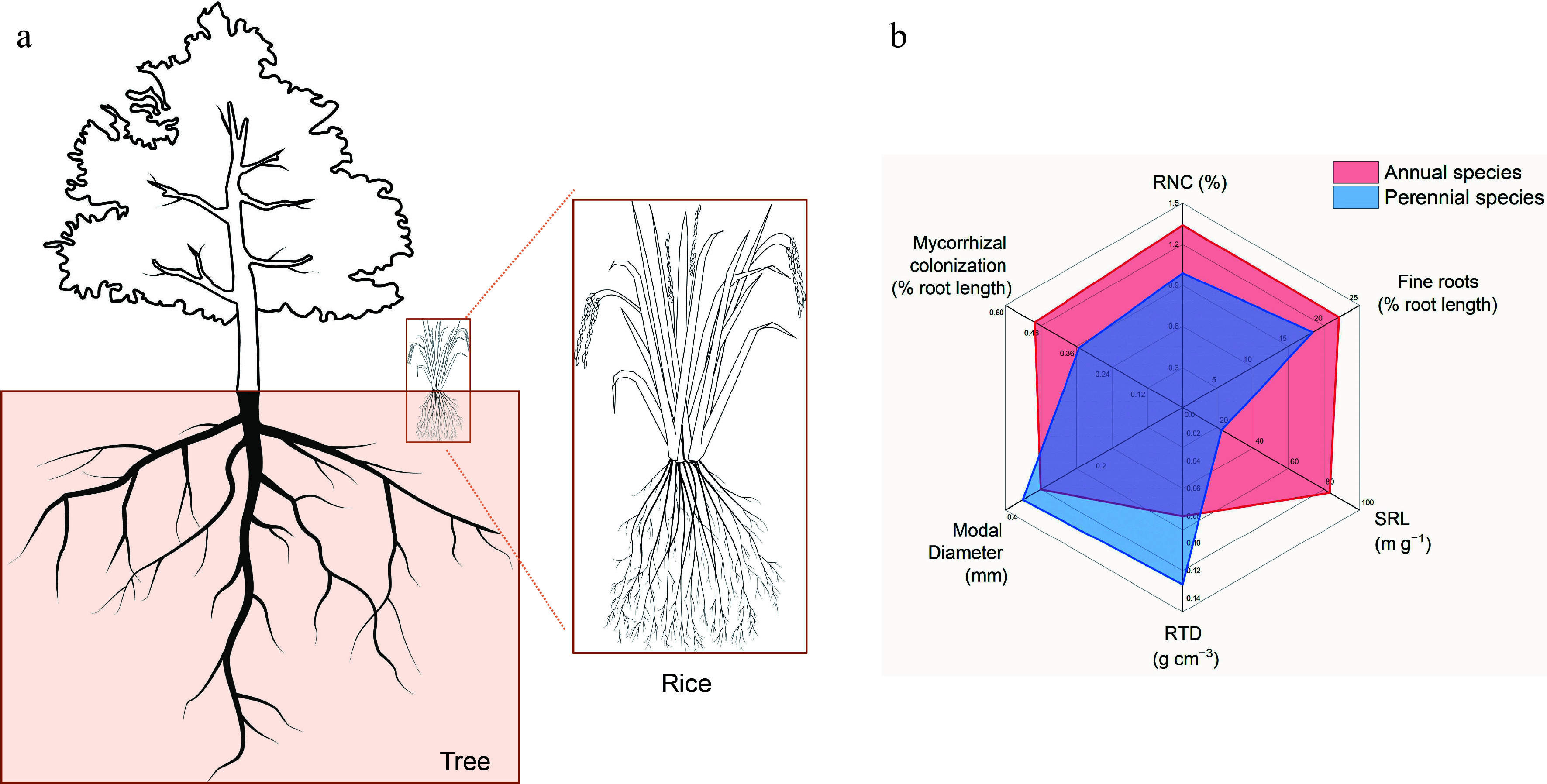
Root system architecture in perennial trees and annual crops. (a) Schematic diagram of RSA in trees and rice. (b) Root traits related to nitrogen absorption in annual species and perennial species according to the data of 18 field-grown species^[25]^. RNC: root nitrogen concentration; Fine roots: proportion of root length with diameter < 0.2 mm; SRL: specific root length; RTD: root tissue density; modal diameter, the size of the diameter that occurs most.

The RSA diverges markedly between perennial trees and annual crops, generating contrasting nitrogen-acquisition strategies. Consistent with Fitter's topological model, perennial trees typically possess a herringbone topology, characterized by a dominant central axis with laterals branching at wide angles^[23]^. This structure allows for extensive soil exploration with minimal construction cost per unit length. Annual crops such as rice, by contrast, elaborate a dichotomous RSA that rapidly proliferates fine roots within discrete soil horizons, yielding significantly higher root length density (RLD) and root mass density (RMD)^[24]^. The resultant amplification of absorptive surface preferentially elevates uptake of both mobile (nitrate) and less-mobile (ammonium) nutrients, thereby sustaining the accelerated growth rates characteristic of short-season crops (Fig. 2a).

Beyond topology, annuals and perennials also differ in several root traits crucial for resource uptake and conservation. Annuals exhibit higher specific root length (SRL), fine roots (proportion of root length with diameter < 0.2 mm), root nitrogen concentration (RNC), and mycorrhizal colonization, while having lower root tissue density (RTD) than perennials^[25]^. Perennial woody species invest in thick, dense roots with low SRL and RNC, prioritizing longevity and sustained soil space occupation under chronic competition. Annual crops, conversely, exhibit high SRL and RNC that drive rapid root elongation and nutrient uptake, enabling fast growth and quick resource exploitation within brief, disturbed growing windows^[25]^ (Fig. 2b).

### Root system architecture modification in trees

The RSA in woody plants is intricately regulated by a complex network of genetic regulators that coordinate processes such as LR formation and primary root elongation. Studies in model tree species like *Populus*, *Malus*, and *Citrus* have identified several key genes and molecular modules central to these developmental processes. For instance, in *Populus*, transcription factors such as PtabZIP1L and PsiSKP2B promote LR growth, while the PtrABR1-PtrYY1 module enhances LR development through downstream targets like *PtrGH3.6* and *PtrPP2C44*^[26−28]^. MicroRNAs also play pivotal roles: miRNA390 stimulates LR formation, whereas miRNA319 negatively regulates LR density by targeting the TCP19-IAA3.2 auxin signaling pathway^[29,30]^. Additionally, *PagWOX11/12a* improves AR development and root elongation, highlighting the involvement of WUSCHEL-related homeobox genes^[31]^. In *Malus*, genes such as *MdARF3* and *MhIDA* peptide positively influence root elongation and LR number, with MdSIZ1 facilitating LR formation via SUMOylation of MdARF8^[32−34]^. The miR156/SPLs module further modulates LR development by repressing NLP7 expression, thereby integrating nitrogen signaling with root morphogenesis^[35]^. These findings underscore a sophisticated genetic framework where transcription factors, small RNAs, and peptide signals converge to fine-tune RSA.

The regulatory mechanisms underlying RSA involve multilayered interactions among transcription factors, hormonal pathways, and post-translational modifications. In *Populus*, PuZFP1 exemplifies a dual-function regulator, inhibiting LR emergence by repressing *PuWRKY46* while promoting AR elongation through suppression of *PuEGR1*, illustrating how single genes can differentially influence root traits^[36]^. Similarly, in *Citrus*, the type-A response regulator CcRR5 interacts with CcRR14 and CcSnRK2s to enhance root length and LR number, linking cytokinin signaling to root development^[37]^. The cooperation between CrWRKY57 and CrABF3 in *Citrus* activates cell cycle gene *CrCYCD6;1*, directly promoting primary root growth and LR proliferation^[38]^. Such combinatorial control is also evident in *Malus*, where MdWOX4-1 activates MdARF3 transcription to regulate LR elongation, while MdARF3 itself modulates the expression of *MdLBD16-2* during distinct LR developmental stages^[32]^. Collectively, these studies reveal that RSA in woody plants is governed by dynamic gene networks, providing a foundational understanding for targeted genetic improvement of root traits (Table 1).

**Table 1:** Key genes of root system architecture regulation in woody plants.

Tree species	Gene	Function	Ref.
*Populus*	*PsiSKP2B*; *PtabZIP1L*; *PeFUS3*; *PtrABR1-PtrYY1* module	Promoting lateral root growth	[26−28,39]
	*MicroRNA319*	Decreasing density of lateral roots	[29]
	*PagWOX11/12a*	Improving adventitious root development; promoting root elongation and biomass	[31,40]
	*MicroRNA390*	Stimulating lateral root development	[30]
	*PuZFP1*	Inhibiting lateral root emergence; promoting adventitious root elongation	[36]
*Malus*	*MdARF3*	Promoting root elongation	[32]
	*MdSIZ1*	Promoting lateral root formation	[34]
	*miR156/SPLs/NLP7* module	Stimulating lateral root development	[35]
	*MhIDA*	Increasing primary root length and lateral root number	[33]
*Citrus*	*CcRR5*	Promoting root length and lateral root number	[37]
	*CrWRKY57; CrABF3*	Increasing primary root length and lateral root number	[38]

### Root system architecture modulation by exogenous nitrogen supply

Low nitrogen availability triggers a conserved foraging program across crops, *Arabidopsis,* and woody species: primary and selected lateral roots elongate, root-to-shoot ratio increases, and longer, finer root hairs proliferate. Collectively, these responses convert a compact root system into an exploratory architecture that maximises soil volume scanned per unit carbon invested. These coordinated morphogenic shifts are orchestrated by local and systemic N signals^[41,42]^.

Mechanistic details of this response remain fragmentary in woody perennials. Time-course transcriptomes of poplar roots under normal and low-N conditions reveal an 11-superhub hierarchy, exemplified by NAC and bZIP members^[43]^. Root-specific up-regulation of *PtaNAC1* increased root biomass and significantly altered the expression of associated hub genes specifically under low-nitrogen conditions in poplar^[44]^. Given the pivotal role of nitrate signaling in root morphogenesis, the sophisticated nitrate-sensing and transduction network delineated in *Arabidopsis* offers a valuable framework for elucidating nitrogen perception and foraging strategies in forest plantations^[45]^.

**Figure 3:**
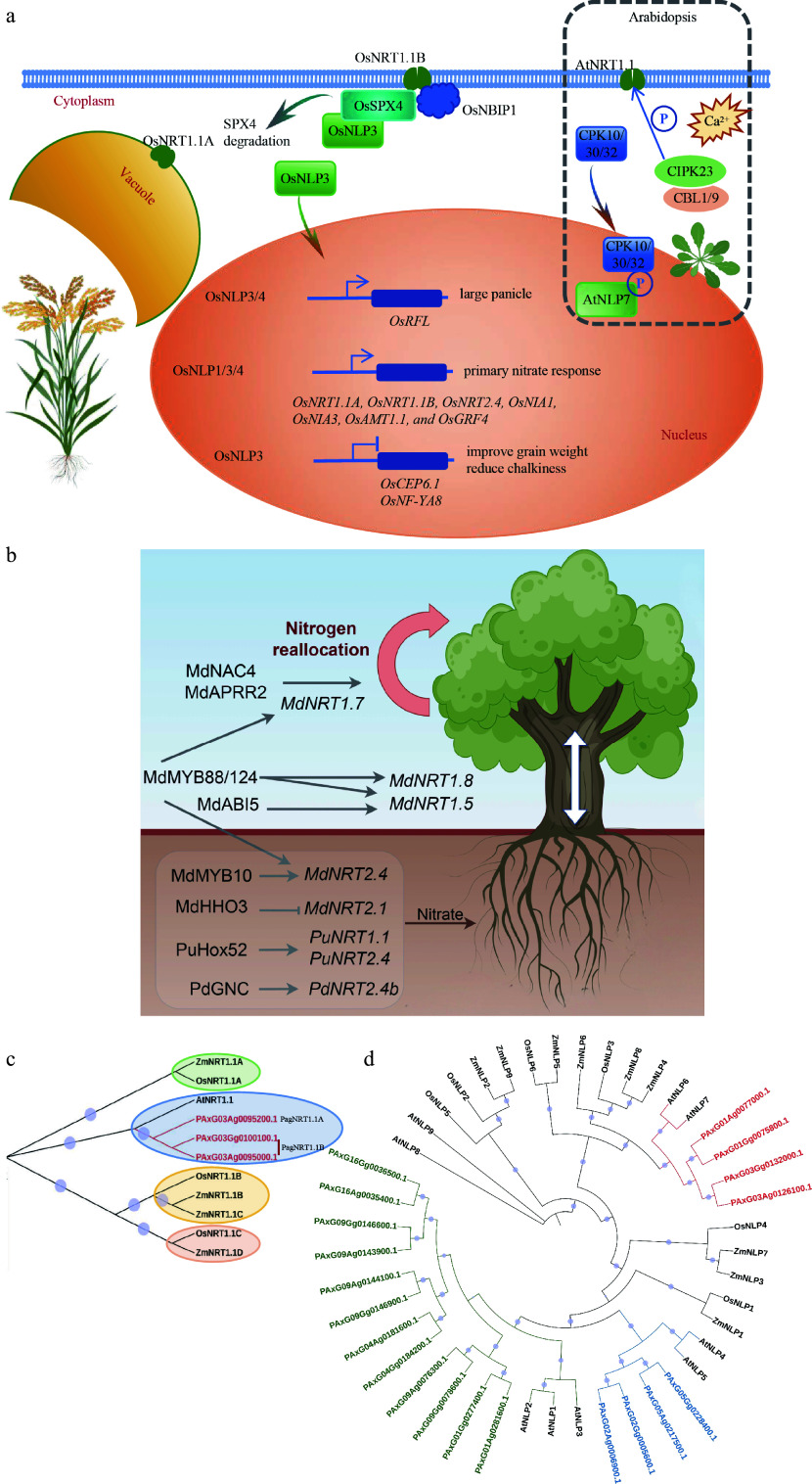
Nitrate sensors function in nutrient uptake and signaling. (a) The role of AtNRT1.1, AtNLP7, and their homologs in rice in nitrate absorption and signal transduction mechanisms. (b) Nitrate transporters and their modulation in poplar and apple trees. (c) Homologous sequence alignment of NRT1.1 in *Arabidopsis*, maize, rice, and poplar 84K. (d) Homologous sequence alignment of NLPs in *Arabidopsis*, maize, rice, and poplar 84K. The phylogenetic tree was constructed using maximum likelihood methods with 5,000 bootstraps in the Tbtools program.

Nitrate, the predominant form of nitrogen available to plants, not only functions as a nutrient but also acts as a signaling molecule that regulates gene expression and triggers responses leading to NUE. In *Arabidopsis thaliana*, the integral membrane protein AtNRT1.1 (also known as CHL1 or NPF6.3) and the transcription factor AtNLP7 have been characterized as key nitrate sensors, playing pivotal roles in the perception and response to nitrate availability^[46,47]^. Loss-of-function mutations in either AtNRT1.1 or AtNLP7 impair nitrate-induced root system remodeling^[47−49]^. Notably, NRT1.1 functions as a dual-affinity nitrate transporter capable of mediating nitrate uptake across a broad concentration range through the modulation by the CBL1/9-CIPK23 complex^[46]^. AtNLP7, a NIN-like protein, accumulates in the nucleus upon phosphorylation by calcium-dependent protein kinase CPK10/30/32 in response to nitrate^[49]^ (Fig. 3a). Intriguingly, recent research in apple trees revealed that the miR156/MdSPL23 module represses nitrate-mediated lateral root development via negative regulation of *MdNLP7*, underscoring the translational potential of key *Arabidopsis*-derived regulatory factors in woody species^[35]^.

### Root system architecture modulation by rhizosphere microorganisms

Rhizosphere microorganisms are increasingly regarded as functional extensions of the plant root system that re-programme RSA via nutrient- and hormone-mediated pathways. In forest plantations, where deeply foraging or highly branched root networks are essential for efficient nitrogen uptake, PGPBs offer an under-exploited lever for RSA engineering. Evidence from poplar indicates that native *Pseudomonas* spp. secrete indole-3-acetic acid (IAA) to stimulate LR proliferation, thereby increasing whole-plant NUE^[50]^. In other tree species, such as *Olea europaea*, *Abies nordmanniana*, *Camellia sinensis,* and *Eucalyptus,* the growth-promoting and nitrogen-contributing functions of various microbial taxa have also been summarized (Table 2). Collectively, these data demonstrate that PGPBs can shape RSA and improve NUE in trees; however, the mechanistic detail lags far behind that available for annual crops.

**Table 2:** Microbes enhancing plant growth and nitrogen use efficiency in forest trees.

Tree species	Bacteria	Function	Ref.
*Populus*	*Pseudomonas*	Enhanced growth, nitrogen acquisition, and secondary root development	[50]
	*Burkholderia* *vietnamiensis*	Nitrogen fixation	[51]
*Olea europaea*	*Azospirillum*, *Bacillus*	Nitrogen fixation, phosphate and potassium solubilization	[52]
*Abies nordmanniana*	*Bacillus*, *Paenibacillus*	Enhanced seed germination, increased secondary root formation	[53]
*Camellia sinensis*	*Klebsiella*, *Serratia*, *Sporosarcina*, *Brevibacillius*	Nitrogen fixation, promote growth, chelate Fe ion	[54]
*Eucalyptus*	*Acidobacteria*, *Verrucomicrobia*, *Chloroflexi*	Nitrogen fixation, Nitrogen cycle	[55]

Phytohormone synthesis is a key mechanism for plant growth promotion by PGPBs, and the mechanisms by which microbial signals regulate RSA in *Arabidopsis*, rice, and maize have been reviewed^[56]^. In maize, nitrogen deficiency induces root exudation of specific flavonoids that enrich *Oxalobacteraceae*; these bacteria, in turn, produce auxin that enhances LR density and improves plant growth under low-N^[57]^. Consistent with this, moderate concentrations of auxin released by *Leifsonia*, *Bacillus*, *Pseudomonas*, and *Serratia* could stimulate root branching and increase root number and biomass^[58−60]^. *Bacillus subtilis* secretes ribosylated cytokinins that stimulate shoot growth and alleviate drought stress in lettuce^[61]^. The gibberellin producers, like *Phoma* *glomerata* and *Penicillium* sp., increased height and biomass in cucumber plants, along with enhanced assimilation of essential^[62,63]^. *Pseudomonas,* which synthesizes gibberellin, was reported to promote the growth of soybean and modulate its growth^[64]^.

Collectively, the data establish a bidirectional nexus: microbiota shape RSA via phytohormone signals, while roots reciprocally sculpt their rhizosphere microbiome through metabolite exudation. Exploiting this dialogue offers a tractable route to engineer RSA and enhance NUE in forest plantations without increasing fertiliser inputs.

## Increasing inorganic nitrogen uptake to enhance tree NUE

Plant N acquisition is tightly coupled to net primary productivity, underscoring the pivotal role of nitrogenous substrates in terrestrial carbon sequestration^[65]^. Globally, plant N acquisition is dominated by inorganic forms, with ammonium and nitrate jointly accounting for 74.4% of total uptake^[66]^. Although the preference for, and rate of, inorganic-N uptake vary markedly among tree species, geographical locations, soil conditions, and even genotypes within a single species, the principal determinants of inorganic-N acquisition efficiency are nitrate influx capacity and ammonium-transporter-mediated NH_4_^+^ uptake^[67−69]^.

### Nitrate uptake and regulatory mechanisms

Nitrate transporters play a key role in both the uptake of nitrate from the external environment and its subsequent internal distribution; 68 NRT/NPF genes have been identified in *P. trichocarpa*^[70]^. Although *in-planta* functions remain insufficiently characterized in forest trees due to a paucity of genetic resources and functional validation, transcriptional regulatory mechanisms governing these transporters have recently advanced in poplar and apple trees (Fig. 3b). In *P. ussuriensis*, the HD-ZIP transcription factor *PuHox52* is specifically induced in roots by N deficiency and directly binds to the promoter of nitrate transporters (*PuNRT1.1*, *PuNRT2.4*, *PuCLC-b*) and nitrate metabolism genes (*PuNIA2*, *PuNIR1*) to improve the NUE^[71]^. Similarly, the GATA transcription factor gene PdGNC also directly modulates the expression of nitrate transporter (*PdNRT2.4b*) and nitrate metabolism genes (*PdNR*, *PdNiR*, and *PdGS2*) to enhance plant growth under nitrogen limitation^[72]^. In *Malus domestica*, *MdNRT2.4-1* could be directly modulated by MdMYB10 to control nitrate uptake and reallocation^[73]^. The repression transcriptional modulation of *MdNRT2.1* by the GARP gene family member, MdHHO3, was also reported^[74]^. Concurrently, additional regulatory factors have been implicated in the long-distance transport of nitrate^[75]^ (Fig. 3b).

Although nitrate acquisition mechanisms have been preliminarily delineated in forest trees, crop studies establish that manipulation of *NRT1.1* or *NLP* genes markedly increases NUE. In rice, the *indica* allele of *OsNRT1.1B* (*OsNPF6.5*) improves NUE by ~30% under low N and ~10% under high N relative to the *japonica* allele^[76]^. Overexpression of the tonoplast-localised *OsNRT1.1A* significantly increases grain yield and shortens the time to maturity^[77]^. Simultaneously, the nitrate-inducible and plasma membrane-localized transporter ZmNRT1.1B plays a crucial role in facilitating root nitrate uptake and signaling, and enhanced expression of *ZmNRT1.1B* significantly increases grain yield under conditions of low to moderate nitrogen availability in field settings in maize^[78]^.

In rice, the function of OsNLP3/4 in the nucleus is contingent upon the degradation of SPX4 mediated by nitrate, which relieves its inhibition on the nuclear import of OsNLP3/4^[79]^. The OsNLP1 protein is nuclear-localized, and its mRNA expression is swiftly up-regulated in response to nitrogen deprivation^[80]^. The impact of OsNLPs on rice yield is not only reflected in the improvement of the efficiency of inorganic nitrogen absorption and utilization but also in the regulation of various aspects such as panicle architecture and grain weight^[81,82]^ (Fig. 3a).

These results establish *NRT1.1* and *NLP* genes as priority targets for breeding crops with optimised nitrogen productivity. To identify the functional orthologs in poplar, a comparative sequence analysis of all NRT and NLP family members was performed across *Arabidopsis*, maize, rice, and poplar, according to the two sets of reference genomes in 84K^[83]^. *PagNRT1.1B* is present as a biallelic locus on both haplotypes, whereas *PagNRT1.1A* occurs on only one haplotype, indicating substantial intra-specific structural variation (Fig. 3c). The NLP family comprises ten members, among which two members exhibit a higher degree of phylogenetic affinity with AtNLP6/NLP7 and OsNLP3, which are considered to be sensor-functioning members. These two members may play a significant role in enhancing nitrogen utilization efficiency in poplar (Fig. 3d). Despite having only two NRT1.1 members and a mere ten NLP family members in poplar 84K, which is not significantly more complex than those in rice and *Arabidopsis*, the functional exploration of their role in NUE remains largely limited.

### Ammonium uptake

In soils where nitrification is suppressed, such as saline-alkaline or water-logged profiles, ammonium becomes the principal nitrogen form acquired by plants and serves as a pivotal nitrogen currency in plant-fungal nutritional exchanges^[84,85]^. Ammonium is transported by ammonium transporters (AMTs) that belong to the AMT/MEP/Rh super-family; the AMT clade is plant-specific, whereas MEP and Rh proteins predominate in yeast and animals, respectively^[86−88]^. In *Arabidopsis*, six *AMT* genes were identified, and high-affinity ammonium uptake is mainly facilitated by AMT1 transporters, with AMT1;1, AMT1;2, AMT1;3, and AMT1;5 contributing 30%, 20%, 30%, and 10%, respectively^[89,90]^. The absence of ammonium transporters can greatly affect the absorption of ammonium by crops, while the overexpression of *AMTs* can improve the growth and yield of crops under low nitrogen conditions^[91,92]^.

However, compared to *Arabidopsis*, the ammonium transport system is highly complex due to the presence of an estimated 14 potential AMTs in poplar trees. Several *AMT*s (*PtAMT1;1*, *PtAMT1;2*, *PtAMT1;5,* and *PtAMT2;2*) show higher expression levels in roots, indicating the potential role in ammonium uptake, and some *AMT*s (*PtAMT1;5*, *PtAMT1;6,* and *PtAMT3;1*) may participate in ammonium reallocation, as the higher expression in mature and senescing leaves than young leaves^[93]^. *PsAMT1.2* overexpression in poplar enhances growth and NUE, whereas ectopic *PtrAMT1;6* expression disrupts carbon-nitrogen balance, diminishes nitrogen assimilation, and ultimately lowers biomass^[94,95]^. Intriguingly, studies in both poplar and *Arabidopsis* reveal that enhancing ammonium uptake efficiency not only elevates NUE but also markedly improves salt tolerance, thereby offering a theoretical foundation for boosting nitrogen utilization in fast-growing plantations established on saline soils^[96,97]^.

AMTs also mediate the transfer of fungal-derived NH_4_^+^ to host plants. In maize (*Zea mays*) colonised by arbuscular mycorrhizal fungi (AMF), *ZmAMT3;1* is specifically induced and plays a primary role in the translocation of ammonium from fungi to maize^[84]^. It is worth noting that an analogous mechanism operates in poplar, where mycorrhizal colonisation specifically up-regulates *PtAMT1;2*, mirroring the AMF-responsive expression of ZmAMT3;1^[84,93]^. Thus, AMTs integrate soil ammonium acquisition with mycorrhizal N delivery, positioning them as dual targets for improving NUE in both fertilised and symbiotic contexts.

## Optimizing the nitrogen cycle to enhance tree NUE

The soil N cycle and its associated microbiota jointly determine the size and turnover rate of the plant-available N pool. In agroecosystems, 30%–60% of applied fertiliser N is lost as NH_3_ volatilisation, NO_3_^-^ leaching or N_2_O emissions, reflecting an inherently low capacity for nitrogen retention^[98]^. Conversely, manipulating microbial N-transforming processes has proved effective in raising crop NUE. In *indica* rice, the root-expressed transceptor NRT1.1B shapes a microbiome enriched in ammonifying taxa; a synthetic community (SynCom) reconstructed from *indica*-enriched isolates accelerates organic-N mineralisation and out-yields a *japonica*-derived SynCom under identical conditions^[99]^. Rhizosphere nitrogen-fixing microorganisms increase and prolong the bioavailability of nitrogen through nitrification, delay flowering by converting tryptophan into the plant hormone 3-Indoleacetic acid (IAA), and also affect vegetative growth by regulating nitrogen utilization^[100]^.

At the biome scale, the nitrogen cycle exhibits a shift from a stable state within forests to a less stable pattern in grasslands, and to an even more dynamic and potentially wasteful process in agricultural lands^[98]^. In recent years, forest research has found that many rhizosphere microorganisms have a significant impact on the nitrogen utilization and nitrogen cycle of trees.

**Figure 4:**
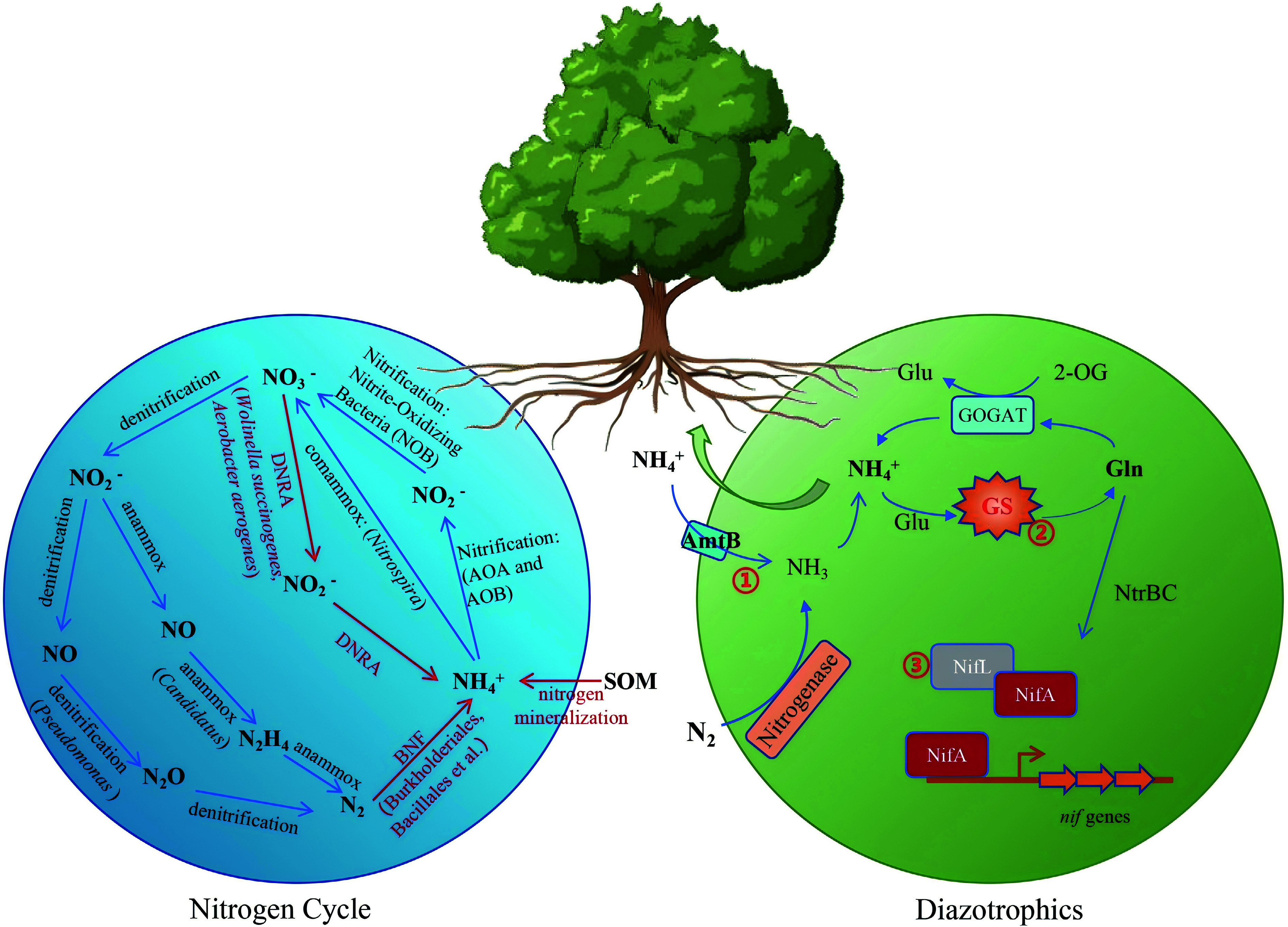
Microbial-mediated nitrogen cycling and BNF enhance NUE in forest trees. The blue circle illustrates various processes of the nitrogen cycle and representative bacterial genera, including denitrification, ammonia oxidation, anammox, and comammox. The green circle represents nitrogen metabolism in associative nitrogen-fixing bacteria and engineering targets for ammonium-excreting strains. (1) Disruption of ammonium transporter AmtB to prevent the ammonium transport back into the cell. (2) Modifying glutamine synthetase to obstruct ammonium assimilation. (3) Modulation of the NifLA system to regulate nitrogenase expression.

BNF is the primary source of new reactive N in unfertilized terrestrial ecosystems and contributes to a major proportion of N-induced new net primary production, up to 3.07 Pg (10^15^g) C yr^−1^, in global forests^[101,102]^. A diverse array of diazotrophs has been identified in the rhizosphere and phyllosphere of forest trees, including Sphingomonadales, Rhizobiales, Pseudomonadales, Burkholderiales, and Bacillales^[103]^. DNRA (dissimilatory nitrate reduction to ammonium), at a rate of 0.24 mg N kg^−1^day^−1^ in forest, conserves nitrogen by converting mobile nitrate into immobile ammonium and prevents losses via denitrification, leaching, and runoff. Representative DNRA-associated microorganisms have been systematically synthesised, such as *Wolinella succinogenes* and *Aerobacter aerogenes*^[104]^. Ammonia-oxidizing archaea (AOA) and ammonia-oxidizing bacteria (AOB) play an important role in nitrification and N_2_O production^[105]^. Studies have demonstrated that nitrification inhibition has the potential to minimize the risk of N loss^[106,107]^. In temperate forest soils, denitrification dominates N_2_ release, accounting for 85.6%–99.5% of total N_2_ production, whereas anammox contributes only 0.5%–14.4%, underscoring its minor role in nitrogen loss via N_2_ emission^[108]^. Four anammox genera have been recognized and named as *Candidatus* Brocadia, *Candidatus* Kuenenia, *Candidatus* Scalindua, and *Candidatus* Anammoxoglobus^[109]^. Soil denitrification is predominantly driven by facultative aerobic heterotrophs, exemplified by *Pseudomonas*, *Bacillus* and *Paracoccus* species, and by the autotrophic bacterium *Thiobacillus denitrificans*^[110]^ (Fig. 4).

Thus, enhancing NUE in forest plantations can therefore be achieved by targeted manipulation of the soil microbiome: promoting BNF and DNRA to channel nitrogen into retained ammonium, while simultaneously suppressing denitrification, nitrification, and anammox to curb nitrogen losses.

## Engineering associated nitrogen-fixation bacteria to enhance tree NUE

Atmospheric dinitrogen (N_2_) constitutes the largest terrestrial N reservoir and is rendered biologically accessible through BNF. This process involves the conversion of atmospheric nitrogen into ammonia or related nitrogenous compounds by certain bacteria and archaea, which can then be utilized by plants and other organisms. It is estimated that the annual global rate of terrestrial biological nitrogen fixation by these organisms is between 52 and 130 Tg of nitrogen per year^[111]^. Legumes (such as soybean, alfalfa, and the wooden-tree black locust, Albizzia) form efficient symbiotic relationships with nitrogen-fixing rhizobia to overcome nitrogen limitations, which is called symbiotic nitrogen fixation (SNF). However, certain non-leguminous plants (such as maize, rice, and the wooden-tree poplar, Scots pine) can also inhabit nutrient-poor environments in the asymbiotic nitrogen fixation (ANF) way by associating with a range of diazotrophic bacteria^[18,51,112]^.

Although nitrogen fixation rate by free-living organisms is significantly lower than that of SNF, ANF contributes substantially to global nitrogen fixation, accounting for at least one-third of the overall terrestrial nitrogen fixation, due to the diversity of diazotrophic organisms across various ecosystems^[111]^. Diazotrophic consortia, which are cooperative assemblages of nitrogen-fixing microbes, colonize plant roots and enhance BNF while supplying the host with fixed nitrogen. These consortia include a wide range of diazotrophs identified in the soil, litter, the rhizosphere and phyllosphere of trees, grasses and crops, as well as in bryophytes and lichen^[113]^. Although ANF constitutes a large proportion of ecosystem-level nitrogen inputs in forests, the inherently low nitrogenase efficiency of natural diazotrophs has long constrained its practical value. Recent advances in the metabolic engineering of associative diazotrophs, initially demonstrated and refined in agricultural systems, have markedly elevated nitrogen fixation rates and host plant benefits, thereby providing a clear technological template for enhancing ANF performance in forest plantations.

In ANF, fixed nitrogen is primarily used by the diazotrophs for their own growth, rather than altruistically shared with plants until becoming available to plants through microbial decomposition^[114,115]^. When an ammonium-releasing strain of *Azospirillum brasilense* was applied as the inoculant to roots of the model C4 grass *Setaria viridis*, it increased the plant nitrogen uptake, as well as the plant height, weight, lateral root number, and root length. Studies utilizing ^13^N_2_ isotope as a tracer have yielded conclusive evidence of its uptake by the host plant, followed by its subsequent integration into the plant's protein structures from bacterial N_2_ fixation^[116]^.

Recent studies have utilized genetic manipulations to interfere with ammonium assimilation or transportation, to block nitrogen fixation repression, or to alter other metabolic processes involved in nitrogen fixation, which could also lead to ammonium excretion. Modifying glutamine synthetase (GS) to obstruct ammonium assimilation was accomplished in several bacteria, resulting in ammonium excretion. Mutants of GS include the GS-P347L mutant of *A. brasilense*, the GS-D49S mutant of *A. vinelandii*, and the GS-Y183C mutant of *A. variabilis*, which have been reported to promote growth and weight of wheat, cucumber, and other plants^[117−119]^. Disruption of ammonium transporter AmtB also prevents the bacteria from transporting ammonium back into the cell in time under the condition of ammonium leakage, thus resulting in the ammonium excretion. As mentioned earlier, nitrogenase transcription in all the Proteobacterial diazotrophs requires the transcriptional activator NifA. The *nifA* gene is located downstream of and cotranscribed with *nifL* in the representative Proteobacterial diazotrophs *K. pneumoniae* and *A. vinelandii*, which constitutes the NifLA system and further regulates nitrogenase expression. The NifLA system can be disrupted by impairing or eliminating NifL, or by overexpressing *NifA*, and its disruption may further lead to ammonium secretion. Furthermore, to bypass the native ammonium-repression feedback of nitrogen fixation, several attempts have been made to transfer *nif* clusters into non-diazotrophic soil or rhizosphere-associated bacteria (e.g., *Pseudomonas protegens*, *Bacillus subtilis*, etc.) and express nitrogenase in a heterologous manner^[120,121]^ (Fig. 4). A recent study has isolated an aerobic nitrogen-fixing endophyte, *Burkholderia vietnamiensis,* from *Populus trichocarpa,* and the modification of these strains with ammonia-secreting characteristics might be a potentially effective method for improving the NUE of poplar trees^[51]^.

## Perspectives for the future

### Enhancing functional validation of key NUE genes through CRISPR-based editing in forest plantations

Future efforts must leverage CRISPR/Cas-based gene editing, preferably DNA-free delivery^[122,123]^, to systematically validate candidate NUE genes in forest plantations under field-relevant nitrogen regimes. While orthologs of AtNRT1.1 and AtNLP7 have been identified in poplar (PagNRT1.1A/B and PagNLPs), their roles in nitrate uptake, long-distance transport, and remobilization remain largely uncharacterized under field-relevant conditions. Beyond nitrate, ammonium transporters such as *PtAMT1;2*, which is up-regulated under mycorrhizal colonization^[93]^, should be targeted to assess their role in ammonium acquisition and redistribution. Moreover, elucidating how xylem-expressed genes react to nitrogen supply and the specific functions of nitrate transporters in long-distance transport will establish a foundation for dissecting their regulatory functions in tree nitrogen metabolism and reallocation, especially for timber purposes^[11]^. Finally, integrating CRISPR screens with single-cell RNA-seq or spatial transcriptomics could reveal nitrogen-responsive gene networks across root zones and vascular tissues, offering a systems-level understanding of NUE in trees.

**Figure 5:**
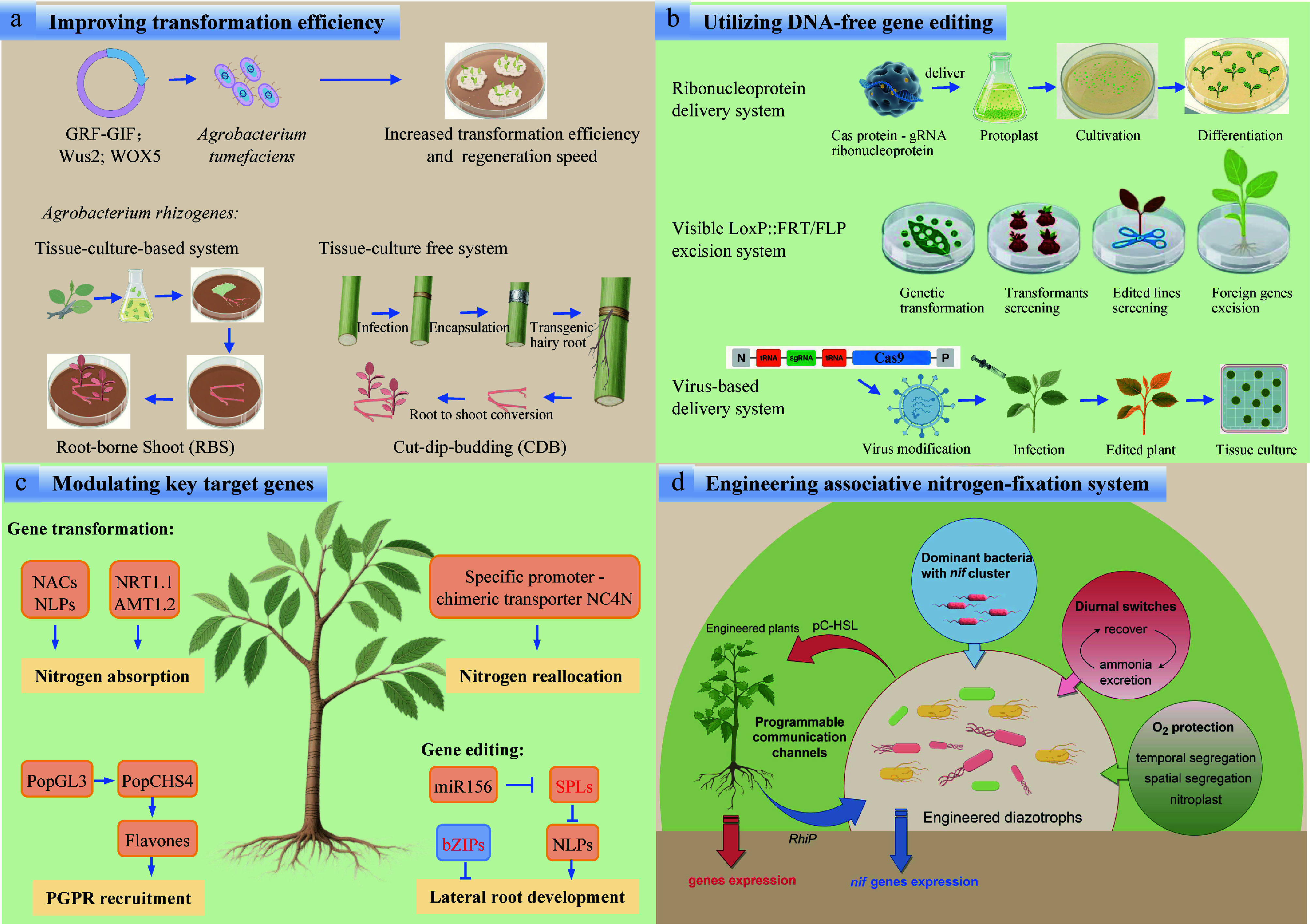
Challenges and measures for improving NUE in forest plantations. (a) Improving transformation efficiency. (b) Utilizing DNA-free gene editing. (c) Modulating key target genes. (d) Engineering associative nitrogen-fixation system.

To directly enhance NUE in forest plantations via gene editing, key targets can be selected among genes governing root development and nitrogen utilization. Studies in crops and forest trees have converged on functionally analogous candidate genes that serve as preferred editing targets for this purpose. MicroRNAs are pivotal regulators of low-nitrogen responses, and their suppression enhances nitrogen-use efficiency in crops. Notably, miR156 is induced by low nitrogen and targets *SPL* genes to improve root development and NUE in rice, sugar beet, alfalfa, moso bamboo, and apple trees^[35,124−127]^. In addition, several transcription factors negatively modulate NUE. Suppression of the bZIP family member *OsbZIP1* in rice promotes root development and elevates NUE, and analogous bZIP genes in poplar exert negative control over root growth. Mining and editing these orthologues, therefore, offer a promising route to directly enhance NUE in forest plantations^[128−130]^ (Fig. 5).

Although gene-editing systems in woody plants have been constrained by low transformation efficiency, high chimerism, and potential off-target effects, substantial progress has nonetheless been achieved in forest tree genome editing^[131]^. The co-expressing developmental regulators, such as *Wus2*, *WOX5*, and GRF-GIF chimeric protein, have been shown to significantly boost regeneration and transformation efficiencies across diverse plant species^[132]^. When combined with *Rhizobium rhizogenes*-mediated hairy-root induction followed by shoot conversion, it provides a streamlined route for genetic transformation in fruit and forest trees, operating effectively in both tissue-culture-based and tissue-culture-free systems^[133,134]^. To eliminate continuous off-target concerns and bypass GMO regulatory hurdles, DNA-free genome editing has been successfully implemented by a LoxP::FRT excision system or by delivering CRISPR/Cas9 ribonucleoprotein complexes (RNPs) in poplar and larch^[122,123]^. In the future, virus-mediated DNA-free delivery systems are expected to substantially overcome current bottlenecks of low delivery efficiency and recalcitrant genotype transformation (Fig. 5).

### Synthetic biology strategies to augment NUE in forest plantations

In recent years, plant synthetic biology, a nascent interdisciplinary field, has merged engineering concepts with plant biology to create and manufacture novel devices^[135]^. Synthetic biology approaches have demonstrated tremendous potential in enhancing the nutrient utilization efficiency of plants. For example, domain-shuffling between AtCHL1(NRT1.1) and AtNRT1.2 generated a chimeric transporter (NC4N) that exhibits hyperactive low-affinity nitrate uptake. When expressed specifically in the phloem of minor veins in senescing leaves with the promoter of AtNRT1.7, the transgenic plants showed enhanced growth or yield in *Arabidopsis*, tobacco, and rice^[136]^.

Equally critical is promoter choice. For example, under the constitutive Ubi promoter, *OsNRT2.1* was ectopically overexpressed 7.5-fold across tissues, yet NUE dropped to 83% of the wild-type. In sharp contrast, *pOsNAR2.1* promoter drove a modest 80% increase in *OsNRT2.1* expression, which translated into a 28% gain in ANUE relative to the wild-type^[137]^. A parallel lesson emerges in poplar, where strong constitutive expression of *PtrAMT1;6* disrupts C-N balance and reduces biomass^[94]^. Together, these studies underscore that promoter strength and cell-type specificity must be co-optimised with transgene function.

Translating these paradigms to forest plantations faces three bottlenecks. First, the nitrogen signalling circuitry of trees is poorly resolved; master transcription factors and post-translational nodes remain orphan. Second, a chassis of nitrogen-responsive, tissue-specific promoters (particularly those active in cambium, ray parenchyma, or phloem-loading zones) is essentially unavailable. Third, long validation cycles preclude rapid empirical optimisation. Machine-learning algorithms and heterologous high-throughput screening will be indispensable to compress development timelines and deliver synthetic NUE traits for plantation forestry^[138]^.

### Engineering diazotrophic consortia and plant-microbe communication to boost NUE

Recent research highlights the potential of synthetic microbial communities (SynComs) to enhance NUE in plants^[99]^. In forest ecosystems, BNF by diazotrophs and DNRA are key processes that channel nitrogen into immobile ammonium, thereby reducing losses via denitrification, leaching, and nitrification. By strategically constructing SynComs enriched with BNF and DNRA microorganisms (such as *Wolinella succinogenes* and *Aerobacter aerogenes*), while simultaneously incorporating nitrification inhibitors and suppressing denitrifying and anammox bacteria, the nitrogen cycle can be steered towards ammonium conservation. This tailored microbial management promotes a more closed-loop N economy, ultimately improving nitrogen acquisition and NUE in plantation forests.

One of the major challenges in engineering the ammonium-tolerant and -secreting diazotrophic strains is ensuring their long-term stability, particularly when their glutamine synthetase (GS) activity is compromised due to the nitrogen starvation stress, glutamine auxotrophy, and energetic constraints^[139,140]^. A recent study exploited a *glnA* mutant, GS-P95L, in *K. oxytoca* that could result in high levels of ammonia excretion depending on diurnal temperature profiles^[141]^. Inoculant bacteria can struggle to colonize the rhizosphere of target plants due to competition with well-adapted resident microbes shaped by evolutionary pressures over time^[142]^. The engineered epiphyte *Pseudomonas protegens* Pf-5 with *nif* clusters showed great potential to improve the nitrogen flux to cereal crops^[120]^. Most enzymes that catalyze nitrogen fixation are highly sensitive to oxygen. Inspired by *Cyanothece*, the transference of 24 *nif* genes, which are specifically expressed in the dark from the diazotrophic cyanobacterium *Cyanothece* sp. ATCC 51142 to its nondiazotrophic counterpart, *Synechocystis* sp. PCC 6803 has the potential to significantly enhance BNF capabilities^[143]^. Moreover, the newly identified nitrogen fixing symbiosis, nitroplast, represents a significant direction for future research in the bioengineering of BNF^[144,145]^ (Fig. 5).

In poplar, recent research has established an interaction model with the aerobic nitrogen-fixing endophyte *Burkholderia vietnamiensis*^[51]^. Consequently, future efforts could focus on engineering this strain as a chassis for selecting ammonium-excreting variants, offering a promising approach for improving nitrogen cycling in trees. To ensure host specificity, synthetic communication channels, such as the rhizopine (*RhiP*) system in barley and *Azorhizobium caulinodans*^[146]^, could be adapted for poplar. Here, poplar roots engineered to secrete *RhiP* would selectively activate nitrogenase expression in co-inoculated RhiP-responsive diazotrophs. Additionally, quorum-sensing circuits based on p-coumaroyl-homoserine lactone (pC-HSL) could enable bidirectional signaling, allowing plants to modulate bacterial nitrogen fixation based on nitrogen demand^[147]^. It is posited that the bidirectional signaling between plants and microbes will exert a profound influence on BNF (Fig. 5).

### Strategies for improving NUE in forest plantations across contrasting cultivation environments

NUE is a critical determinant of productivity in forest plantations, yet it is severely constrained by site-specific environmental stresses. Therefore, optimization strategies for improving NUE in forest plantations must be tailored to the specific environmental constraints of each site. For example, in cobble and sandy riparian zones, rapid drainage leads to the leaching of nitrate within hours. In this case, introducing engineered, ammonium-excreting *Burkholderia vietnamiensis* can substantially supply nitrogen to plants through BNF. In nitrogen-poor marginal lands or frequently rotated short-rotation plantations, strategies that focus on enhancing LR growth and nitrate uptake capacity by overexpressing transcription factors such as *PtaNAC1*, *OsNLP*s, and nitrate transporters (*OsNRT*s) could be more effective. In nursery and orchard cultivation, excessive nitrogen fertilizer application often leads to lower NUE. In these cases, enhancing LR development by overexpressing *PsiSKP2B*, *PagWOX11/12a,* and *CcRR5* genes in the root system can significantly increase NUE. For the organic-rich zone, promoting fungal colonization and enhancing DNRA can redirect the nitrogen cycle towards more plant-available forms. Moreover, the study of transporters such as AtAMT1;1 and OsNRT1.1B, and their functions in nutrient acquisition, as well as salt and drought stress tolerance, provides a foundation for developing innovative breeding strategies for plantations in saline-alkali and arid environments^[97,148]^ (Table 3).

**Table 3:** Strategies for improving NUE in forest plantations across contrasting cultivation environments.

Plantation conditions	Major limiting factors	Key intervention/target	Expected effect
Cobble-and-sand riparian zone	Extremely low available nitrogen	Engineer ammonium-excreting diazotrophic like *strains Burkholderia vietnamiensis*	Enhance BNF-derived N supply
Nitrogen-poor marginal land	Low concentration of nitrogen and shallow root	Mixed forest with N_2_-fixing tree species; overexpress *PsiSKP2B*, *PagWOX11/12a* and *CcRR5* genes	Enhance the input of available nitrogen; improve root elongation and lateral-root development
Frequent rotation	Nitrogen depletion	Overexpress *PuHox52*, *PtaNAC1*, *PdGNC*, *OsNLP*s and *OsNRT*s	Boost lateral-root development and N-uptake efficiency
Organic-rich zone	Low organic-N uptake efficiency; N emission in nitrogen cycle	Enhance fungal colonization; enhance DNRA to retain nitrogen as ammonium	Enhance nitrogen acquisition via fungal pathways; improve soil ammonium content
Nitrogen-rich soil	Restricted lateral-root development; limited nitrogen assimilation rate	Overexpress *PsiSKP2B*, *PagWOX11/12a*, *CcRR5* and *GS* genes	Increase soil-contact area to facilitate nitrogen acquisition; increase nitrogen assimilation rate
Saline-alkaline soil	Suppressed nitrification	Overexpress *PtAMT1;2* or *AtAMT1;1*	Increase ammonium uptake and salt tolerance
Arid region	Decreased soluble nitrogen; decreased *NRT*s gene expression	Overexpress *OsNRT1.1B*	Increase nitrate uptake and drought tolerance

## Data Availability

Data sharing not applicable to this article as no datasets were generated or analyzed during the current study.

## References

[1] (2023). Forest microbiome and global change. Nature Reviews Microbiology.

[2] (2021). Global maps of twenty-first century forest carbon fluxes. Nature Climate Change.

[b3] 3Zalesny RSJr, Barzagli A, Caldwell B, Minotta G, Nervo G, et al. 2025. Innovative practices in the sustainable management of fast-growing trees – Lessons learned from poplars and willows and other experiences with fast-growing trees around the world. Rome, Italy: Food and Agriculture Organization of the United Nations (FAO). doi: 10.4060/cd4104en

[4] (2015). Boreal forest health and global change. Science.

[5] (2006). Carbon partitioning to mobile and structural fractions in poplar wood under elevated CO_2_ (EUROFACE) and N fertilization. Global Change Biology.

[6] (2017). Post-thinning density and fertilization affect *Pinus taeda* stand and individual tree growth. Forest Ecology and Management.

[7] (2013). Fertilizer management of eucalypt plantations on sandy soil in Brazil: Initial growth and nutrient cycling. Forest Ecology and Management.

[8] (2019). Growth performance, photosynthesis, and root characteristics are associated with nitrogen use efficiency in six poplar species. Environmental and Experimental Botany.

[9] (2024). Wood formation in trees responding to nitrogen availability. Industrial Crops and Products.

[10] (2004). Can less yield more? Is reducing nutrient input into the environment compatible with maintaining crop production. Trends in Plant Science.

[11] (2023). Combining GS-assisted GWAS and transcriptome analysis to mine candidate genes for nitrogen utilization efficiency in *Populus cathayana*. BMC Plant Biology.

[12] (2023). Cost-effective mitigation of nitrogen pollution from global croplands. Nature.

[13] (2021). Data on the effects of fertilization on growth rates, biomass allocation, carbohydrates and nutrients of nitrogen-fixing and non-nitrogen-fixing tree legumes during tropical forest restoration. BMC Research Notes.

[14] (2015). Fast growing plantations for wood production – integration of ecological effects and economic perspectives. Frontiers in Bioengineering and Biotechnology.

[15] (2017). Enhanced decomposition and nitrogen mineralization sustain rapid growth of *Eucalyptus regnans* after wildfire. Journal of Ecology.

[16] (2018). Modeling rhizosphere carbon and nitrogen cycling in *Eucalyptus* plantation soil. Biogeosciences.

[17] (2020). Rhizosphere microbiological processes and eucalypt nutrition: Synthesis and conceptualization. Science of The Total Environment.

[18] (2023). Diverse interactions: root-nodule formation and herb-layer composition in black locust (*Robinia pseudoacacia*) stands. Plants.

[19] (2021). Light, nitrogen supply, and neighboring plants dictate costs and benefits of nitrogen fixation for seedlings of a tropical nitrogen-fixing tree. New Phytologist.

[b20] 20Eissenstat DM, Volder A. 2005. The efficiency of nutrient acquisition over the life of a root. In Nutrient Acquisition by Plants: An Ecological Perspective, ed. BassiriRad H. Berlin, Heidelberg: Springer. pp. 185−220 doi: 10.1007/3-540-27675-0_8

[21] (2015). Global poplar root and leaf transcriptomes reveal links between growth and stress responses under nitrogen starvation and excess. Tree Physiology.

[22] (2025). Conservation and divergence of regulatory architecture in nitrate-responsive plant gene circuits. The Plant Cell.

[23] (1987). An architectural approach to the comparative ecology of plant root systems. New Phytologist.

[24] (2009). Desirable plant root traits for protecting natural and engineered slopes against landslides. Plant and Soil.

[25] (2006). Suites of root traits differ between annual and perennial species growing in the field. New Phytologist.

[26] (2017). Poplar *PtabZIP1-like* enhances lateral root formation and biomass growth under drought stress. The Plant Journal.

[27] (2025). Integrating 3D imaging, GWAS and single-cell transcriptome approaches to elucidate root system architecture in *Populus*. Plant Physiology.

[28] (2023). PtrABR1 increases tolerance to drought stress by enhancing lateral root formation in *Populus trichocarpa*. International Journal of Molecular Sciences.

[29] (2025). MicroRNA319-TCP19-IAA3.2 module mediates lateral root growth in *Populus tomentosa*. Plants.

[30] (2018). The *MicroRNA390/TRANS-ACTING SHORT INTERFERING RNA3* module mediates lateral root growth under salt stress via the auxin pathway. Plant Physiology.

[31] (2022). PagWOX11/12a positively regulates the *PagSAUR36* gene that enhances adventitious root development in poplar. Journal of Experimental Botany.

[32] (2024). *MdARF3* switches the lateral root elongation to regulate dwarfing in apple plants. Horticulture Research.

[33] (2025). MhIDA small peptides modulate the growth and development of roots in *Malus hupehensis*. Plant Cell Reports.

[34] (2021). Apple SUMO E3 ligase MdSIZ1 facilitates SUMOylation of MdARF8 to regulate lateral root formation. New Phytologist.

[35] (2025). Involvement of the miR156/SPLs/NLP7 modules in plant lateral root development and nitrogen uptake. Planta.

[36] (2025). PuUBL5-mediated ZINC FINGER PROTEIN 1 stability is critical for root development under drought stress in *Populus ussuriensis*. Plant Physiology.

[37] (2023). CcRR5 interacts with CcRR14 and CcSnRK2s to regulate the root development in citrus. Frontiers in Plant Science.

[38] (2025). CrWRKY57 and CrABF3 cooperatively activate *CrCYCD6;1* to modulate drought tolerance and root development. Horticulture Research.

[39] (2025). PeFUS3 drives lateral root growth via auxin and ABA signalling under drought stress in *populus*. Plant, Cell & Environment.

[40] (2020). WUSCHEL-related homeobox gene *PagWOX11/12a* responds to drought stress by enhancing root elongation and biomass growth in poplar. Journal of Experimental Botany.

[41] (2014). Root nutrient foraging. Plant Physiology.

[42] (2013). Plasticity of the *Arabidopsis* root system under nutrient deficiencies. Plant Physiology.

[43] (2015). A systems biology approach identifies new regulators of poplar root development under low nitrogen. The Plant Journal.

[44] (2013). Nitrogen deprivation promotes *Populus* root growth through global transcriptome reprogramming and activation of hierarchical genetic networks. New Phytologist.

[45] (2017). Ammonium as a signal for physiological and morphological responses in plants. Journal of Experimental Botany.

[46] (2009). CHL1 functions as a nitrate sensor in plants. Cell.

[47] (2022). NIN-like protein 7 transcription factor is a plant nitrate sensor. Science.

[48] (2006). The *Arabidopsis* NRT1.1 transporter participates in the signaling pathway triggering root colonization of nitrate-rich patches. Proceedings of the National Academy of Sciences of the United States of America.

[49] (2017). Discovery of nitrate–CPK–NLP signalling in central nutrient–growth networks. Nature.

[50] (2025). Flavones enrich rhizosphere *Pseudomonas* to enhance nitrogen utilization and secondary root growth in *Populus*. Nature Communications.

[51] (2024). Dynamic nitrogen fixation in an aerobic endophyte of *Populus*. The ISME Journal.

[52] (2020). The role of microbial inoculants on plant protection, growth stimulation, and crop productivity of the olive tree (*Olea europea* L.). Plants.

[53] (2020). Identification of root-associated bacteria that influence plant physiology, increase seed germination, or promote growth of the christmas tree species *Abies nordmanniana*. Frontiers in Microbiology.

[54] (2023). A review on rhizosphere microbiota of tea plant (*Camellia sinensis* L): recent insights and future perspectives. Journal of Agricultural and Food Chemistry.

[55] (2022). Impacts of near-natural management in eucalyptus plantations on soil bacterial community assembly and function related to nitrogen cycling. Functional Ecology.

[56] (2024). Signal communication during microbial modulation of root system architecture. Journal of Experimental Botany.

[57] (2021). Plant flavones enrich rhizosphere Oxalobacteraceae to improve maize performance under nitrogen deprivation. Nature Plants.

[58] (2017). Effect of auxin producing and phosphate solubilizing bacteria on mobility of soil phosphorus, growth rate, and P acquisition by wheat plants. Acta Physiologiae Plantarum.

[59] (2016). Association of plant growth-promoting *Serratia* spp. with the root nodules of chickpea. Research in Microbiology.

[60] (2016). Differential effects of plant growth-promoting rhizobacteria on maize growth and cadmium uptake. Journal of Plant Growth Regulation.

[61] (2007). Cytokinin producing bacteria enhance plant growth in drying soil. Plant and Soil.

[62] (2012). Endophytic fungi produce gibberellins and indoleacetic acid and promotes host-plant growth during stress. Molecules.

[63] (2018). Gibberellin biosynthesis and metabolism: a convergent route for plants, fungi and bacteria. Microbiological Research.

[64] (2014). Gibberellin secreting rhizobacterium, *Pseudomonas putida* H-2-3 modulates the hormonal and stress physiology of soybean to improve the plant growth under saline and drought conditions. Plant Physiology and Biochemistry.

[65] (2006). Nitrogen uptake, distribution, turnover, and efficiency of use in a CO_2_-enriched sweetgum forest. Ecology.

[66] (2025). Plant nitrogen uptake preference and drivers in natural ecosystems at the global scale. New Phytologist.

[67] (2005). Climate and forest management influence nitrogen balance of European beech forests: microbial N transformations and inorganic N net uptake capacity of mycorrhizal roots. European Journal of Forest Research.

[68] (2005). Soil retention, tree uptake, and tree resorption of ^15^NH_4_NO_3_ and NH_4_^15^NO_3_ applied to trembling and hybrid aspens at planting. Canadian Journal of Forest Research.

[69] (2025). Tree root nutrient uptake kinetics vary with nutrient availability, environmental conditions, and root traits: a global analysis. New Phytologist.

[70] (2013). The nitrate transporter (NRT) gene family in poplar. PLoS ONE.

[71] (2023). PuHox52 promotes coordinated uptake of nitrate, phosphate, and iron under nitrogen deficiency in *Populus ussuriensis*. Journal of Integrative Plant Biology.

[72] (2022). The transcription factor GNC optimizes nitrogen use efficiency and growth by up-regulating the expression of nitrate uptake and assimilation genes in poplar. Journal of Experimental Botany.

[73] (2022). MdMYB10 affects nitrogen uptake and reallocation by regulating the nitrate transporter MdNRT2.4-1 in red-fleshed apple. Horticulture Research.

[74] (2022). The apple GARP family gene MdHHO3 regulates the nitrate response and leaf senescence. Frontiers in Plant Science.

[75] (2022). MdNAC4 interacts with MdAPRR2 to regulate nitrogen deficiency-induced leaf senescence in apple (*Malus domestica*). Frontiers in Plant Science.

[76] (2015). Variation in NRT1.1B contributes to nitrate-use divergence between rice subspecies. Nature Genetics.

[77] (2018). Expression of the nitrate transporter gene *OsNRT1.1A/OsNPF6.3* confers high yield and early maturation in rice. The Plant Cell.

[78] (2024). ZmNRT1.1B (ZmNPF6.6) determines nitrogen use efficiency via regulation of nitrate transport and signalling in maize. Plant Biotechnology Journal.

[79] (2019). Nitrate–NRT1.1B–SPX4 cascade integrates nitrogen and phosphorus signalling networks in plants. Nature Plants.

[80] (2020). Rice NIN-LIKE PROTEIN 1 rapidly responds to nitrogen deficiency and improves yield and nitrogen use efficiency. Journal of Experimental Botany.

[81] (2024). OsNLP3 enhances grain weight and reduces grain chalkiness in rice. Plant Communications.

[82] (2023). The OsNLP3/4-OsRFL module regulates nitrogen-promoted panicle architecture in rice. New Phytologist.

[83] (2024). High-quality genome assembly enables prediction of allele-specific gene expression in hybrid poplar. Plant Physiology.

[84] (2022). The mycorrhiza-specific ammonium transporter ZmAMT3;1 mediates mycorrhiza-dependent nitrogen uptake in maize roots. The Plant Cell.

[85] (2002). NH4+ toxicity in higher plants: a critical review. Journal of Plant Physiology.

[86] (2005). Evolutionary conservation and diversification of Rh family genes and proteins. Proceedings of the National Academy of Sciences of the United States of America.

[87] (2001). Rhesus factors and ammonium: a function in efflux?. Genome Biology.

[88] (2012). Multiple horizontal gene transfers of ammonium transporters/ammonia permeases from prokaryotes to eukaryotes: toward a new functional and evolutionary classification. Molecular Biology and Evolution.

[89] (2007). The organization of high-affinity ammonium uptake in *Arabidopsis* roots depends on the spatial arrangement and biochemical properties of AMT1-type transporters. The Plant Cell.

[90] (2020). CALCIUM-DEPENDENT PROTEIN KINASE 32-mediated phosphorylation is essential for the ammonium transport activity of AMT1;1 in Arabidopsis roots. Journal of Experimental Botany.

[91] (2022). OsAMT1;1 and OsAMT1;2 coordinate root morphological and physiological responses to ammonium for efficient nitrogen foraging in rice. Plant and Cell Physiology.

[92] (2023). Ectopic expression of sugarcane *ScAMT1.1* has the potential to improve ammonium assimilation and grain yield in transgenic rice under low nitrogen stress. International Journal of Molecular Sciences.

[93] (2007). The expanded family of ammonium transporters in the perennial poplar plant. New Phytologist.

[94] (2023). Functional identification and genetic transformation of the ammonium transporter *PtrAMT1;6* in *Populus*. International Journal of Molecular Sciences.

[95] (2021). Ammonium transporter PsAMT1.2 from *Populus simonii* functions in nitrogen uptake and salt resistance. Tree Physiology.

[96] (2024). Physiological and transcriptomic analyses reveal the molecular mechanism of *PsAMT1.2* in salt tolerance. Tree Physiology.

[97] (2025). SALT OVERLY SENSITIVE2 and AMMONIUM TRANSPORTER1;1 contribute to plant salt tolerance by maintaining ammonium uptake. The Plant Cell.

[98] (2023). Expanding agroforestry can increase nitrate retention and mitigate the global impact of a leaky nitrogen cycle in croplands. Nature Food.

[99] (2019). NRT1.1B is associated with root microbiota composition and nitrogen use in field-grown rice. Nature Biotechnology.

[100] (2018). Rhizosphere microorganisms can influence the timing of plant flowering. Microbiome.

[101] (2013). Biological nitrogen fixation: rates, patterns and ecological controls in terrestrial ecosystems. Philosophical Transactions of the Royal Society B: Biological Sciences.

[102] (2018). Nitrogen-induced new net primary production and carbon sequestration in global forests. Environmental Pollution.

[103] (2023). Harnessing biological nitrogen fixation in plant leaves. Trends in Plant Science.

[104] (2020). DNRA: a short-circuit in biological N-cycling to conserve nitrogen in terrestrial ecosystems. Science of The Total Environment.

[105] (2016). Effects of the nitrification inhibitor 3, 4-dimethylpyrazole phosphate on nitrification and nitrifiers in two contrasting agricultural soils. Applied and Environmental Microbiology.

[106] (2008). Detection, isolation and characterization of a root-exuded compound, methyl 3-(4-hydroxyphenyl) propionate, responsible for biological nitrification inhibition by sorghum (*Sorghum bicolor*). New Phytologist.

[107] (2016). Biological nitrification inhibition by rice root exudates and its relationship with nitrogen-use efficiency. New Phytologist.

[108] (2016). Contribution of anammox to nitrogen removal in two temperate forest soils. Applied and Environmental Microbiology.

[109] (2008). *Candidatus* 'Brocadia fulgida': an autofluorescent anaerobic ammonium oxidizing bacterium. FEMS Microbiology Ecology.

[110] (2016). Ecology of nitrogen fixing, nitrifying, and denitrifying microorganisms in tropical forest soils. Frontiers in Microbiology.

[111] (2020). The global distribution of biological nitrogen fixation in terrestrial natural ecosystems. Global Biogeochemical Cycles.

[112] (2023). Presence and activity of nitrogen-fixing bacteria in Scots pine needles in a boreal forest: a nitrogen-addition experiment. Tree Physiology.

[113] (2011). Functional ecology of free-living nitrogen fixation: a contemporary perspective. Annual Review of Ecology, Evolution, and Systematics.

[114] (2018). Nitrogen fixation in cereals. Frontiers in Microbiology.

[115] (2019). To fix or not to fix: controls on free-living nitrogen fixation in the rhizosphere. Applied and Environmental Microbiology.

[116] (2015). Robust biological nitrogen fixation in a model grass–bacterial association. The Plant Journal.

[117] (2017). Metabolic engineering of a diazotrophic bacterium improves ammonium release and biofertilization of plants and microalgae. Metabolic Engineering.

[118] (2003). Altered kinetic properties of tyrosine-183 to cysteine mutation in glutamine synthetase of *Anabaena variabilis* strain SA1 is responsible for excretion of ammonium ion produced by nitrogenase. Current Microbiology.

[119] (2017). Wheat colonization by an *Azospirillum brasilense* ammonium-excreting strain reveals upregulation of nitrogenase and superior plant growth promotion. Plant and Soil.

[120] (2020). Control of nitrogen fixation in bacteria that associate with cereals. Nature Microbiology.

[121] (2018). Engineered integrative and conjugative elements for efficient and inducible DNA transfer to undomesticated bacteria. Nature Microbiology.

[122] (2025). A visual monitoring DNA-free multi-gene editing system excised via LoxP::FRT/FLP in poplar. Plant Biotechnology Journal.

[123] (2024). CRISPR/Cas9 ribonucleoprotein mediated DNA-free genome editing in larch. Forestry Research.

[124] (2023). Identification and characterization of CircRNA-associated CeRNA networks in moso bamboo under nitrogen stress. BMC Plant Biology.

[125] (2024). MicroRNAs participate in morphological acclimation of sugar beet roots to nitrogen deficiency. International Journal of Molecular Sciences.

[126] (2012). Identification and comparative analysis of microRNAs associated with low-N tolerance in rice genotypes. PLoS One.

[127] (2017). MsmiR156 affects global gene expression and promotes root regenerative capacity and nitrogen fixation activity in alfalfa. Transgenic Research.

[128] (2021). Mutation of OUR1/OsbZIP1, which encodes a member of the basic leucine zipper transcription factor family, promotes root development in rice through repressing auxin signaling. Plant Science.

[129] (2024). OsbZIP1 regulates phosphorus uptake and nitrogen utilization, contributing to improved yield. The Plant Journal.

[130] (2020). The *bZIP53–IAA4* module inhibits adventitious root development in Populus. Journal of Experimental Botany.

[131] (2022). Progress and challenges in applying CRISPR/Cas techniques to the genome editing of trees. Forestry Research.

[132] (2020). A GRF–GIF chimeric protein improves the regeneration efficiency of transgenic plants. Nature Biotechnology.

[133] (2024). An efficient genetic transformation system mediated by *Rhizobium rhizogenes* in fruit trees based on the transgenic hairy root to shoot conversion. Plant Biotechnology Journal.

[134] (2025). Establishment of an efficient *Agrobacterium rhizogenes*-mediated hairy root transformation method for subtropical fruit trees. Horticultural Plant Journal.

[135] (2015). Plant synthetic biology. Trends in Plant Science.

[136] (2020). Improving nitrogen use efficiency by manipulating nitrate remobilization in plants. Nature Plants.

[137] (2016). Agronomic nitrogen-use efficiency of rice can be increased by driving *OsNRT2.1* expression with the *OsNAR2.1* promoter. Plant Biotechnology Journal.

[138] (2023). Making small molecules in plants: a chassis for synthetic biology-based production of plant natural products. Journal of Integrative Plant Biology.

[139] (2015). Gene deletions resulting in increased nitrogen release by *Azotobacter vinelandii*: application of a novel nitrogen biosensor. Applied and Environmental Microbiology.

[140] (2020). Harnessing atmospheric nitrogen for cereal crop production. Current Opinion in Biotechnology.

[141] (2023). Diurnal switches in diazotrophic lifestyle increase nitrogen contribution to cereals. Nature Communications.

[142] (2021). Engineering rhizobacteria for sustainable agriculture. The ISME Journal.

[143] (2018). Engineering nitrogen fixation activity in an oxygenic phototroph. mBio.

[144] (2024). Nitrogen-fixing organelle in a marine alga. Science.

[145] (2024). Metabolic trade-offs constrain the cell size ratio in a nitrogen-fixing symbiosis. Cell.

[146] (2022). Engineered plant control of associative nitrogen fixation. Proceedings of the National Academy of Sciences of the United States of America.

[147] (2024). Synthetic microbe-to-plant communication channels. Nature Communications.

[148] (2025). NRT1.1B acts as an abscisic acid receptor in integrating compound environmental cues for plants. Cell.

